# Otorrhea as a Resolution for a Parotid Abscess through an Unusual Natural Pathway: A Case Report and Recent Literature Review

**DOI:** 10.22038/IJORL.2021.58379.3014

**Published:** 2022-03

**Authors:** Suhaili Zazali, Senthilraj Retinasekharan

**Affiliations:** 1 *Department of Otorhinolaryngology - Head and Neck Surgery, Kulliyah of Medicine, International Islamic University Malaysia, Bandar Indera Mahkota Campus, Jalan Sultan Ahmad Shah, 25200 Kuantan, Pahang.*; 2 *Department of Otorhinolaryngology Head and Neck Surgery, Hospital Shah Alam, Persiaran Kayangan, Seksyen 7, 40000 Shah Alam, Selangor, Malaysia. *

**Keywords:** Parotid abscess, Otorrhea, Fissure of Santorini

## Abstract

**Introduction:**

The connection between parotid gland and external auditory canal has long been described throughout the centuries. It can act as a gateway for infectious or neoplastic material to spread between those two structures.

**Case Report:**

To our surprise, this naturally occurring defect can serve as an option to conservatively treat a parotid abscess. We report a case of a parotid abscess with a concurrent presentation of an ipsilateral ear discharge. The purpose of our study is to highlight a unique process of resolution of parotid abscess through an opening in the ear canal.

**Conclusions:**

In spite of the fact that the fissure of Santorini is known as the gateway and tunnel for a disease to spread, it has proven to serve as a pathway for disease elimination as well.

## Introduction

The fissures of Santorini are horizontal openings in the anteroinferior cartilaginous part of the ear canal. These fissures render more flexibility to the ear canal but as they are connected to the parotid gland, they can allow bacteria and tumours to pass into the gland (1). Although its anatomical existence has long been discovered, disease extensions from the external auditory canal to the parotid gland are still being reported as a rare finding. It is even rarer for the spread of disease to occur in the opposite direction – from the parotid to the ear (2). The present case described a parotid abscess in association with an underlying sialolithiasis and an ipsilateral ear discharge presentation. Attention needs to be drawn to the mechanism behind the resolution of abscess by means of otorrhea. 

## Case Report

**Fig 1:**
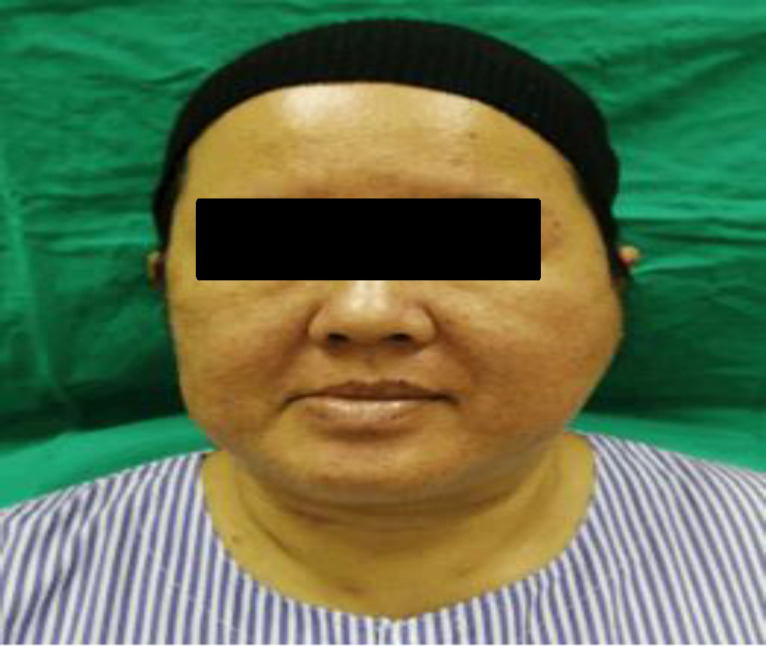
Unilateral left parotid swelling with normal skin appearance

A 40-year-old female at 4 weeks’ gestation presented with left parotid swelling followed by left ear pululent discharge five days later. The swelling was associated with pain and limited mouth opening. On examination, the patient was well, comfortable with documented normal temperature. There was a 9 cm x 6 cm, firm, tender swelling over the left parotid with a normal overlying skin (Figure 1). Her facial nerve was intact. 

**Fig 2:**
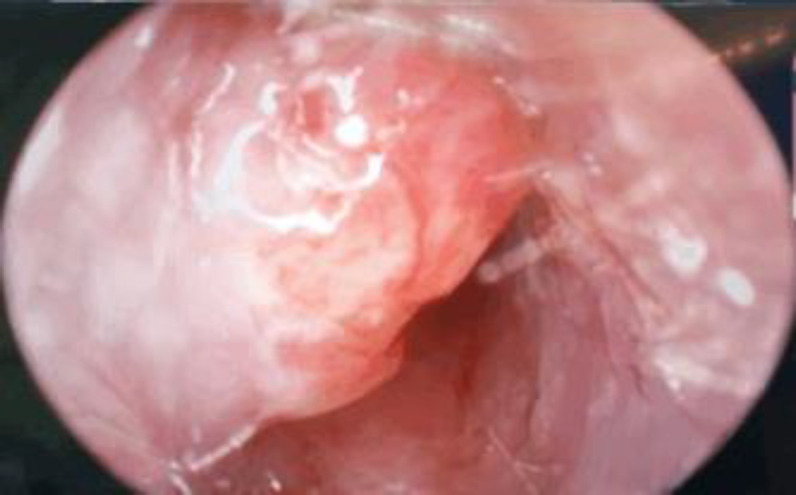
Granulation tissue at the anterior cartilaginous wall of left external auditory canal

**Fig 3:**
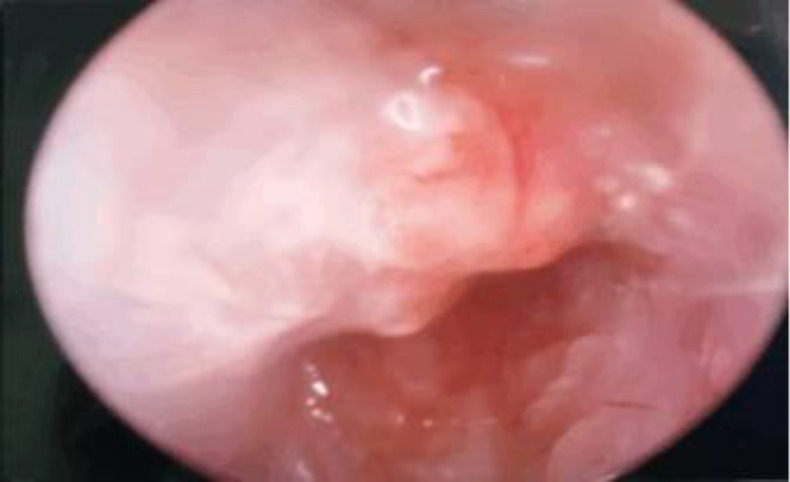
Discharge draining on the granulation tissue upon external pressure of the parotid gland

Otoscopic examination of the left ear revealed a granulation tissue over the anterior wall of the cartilaginous part of the ear canal (Figure 2). Pus was noted to be draining from below the granulation tissue upon application of pressure to the parotid region (Figure 3). 

Oral cavity examination revealed a poor oral hygiene with multiple dental caries. There was no discharge from the Stensen's duct. She required admission and started on intravenous amoxicillin/clavulanic acid 1200mg 8 hourly. As she was in her early trimester, non-invasive investigations were chosen. Ultrasound showed a heterogeneous collection seen at the left parotid measuring 1.2 cm x3.7 cm x 3.1 cm (AP x W x CC). There was an echogenic structure within the collection measuring 0.7 cm (W) suggestive of calculus. 

**Fig 4:**
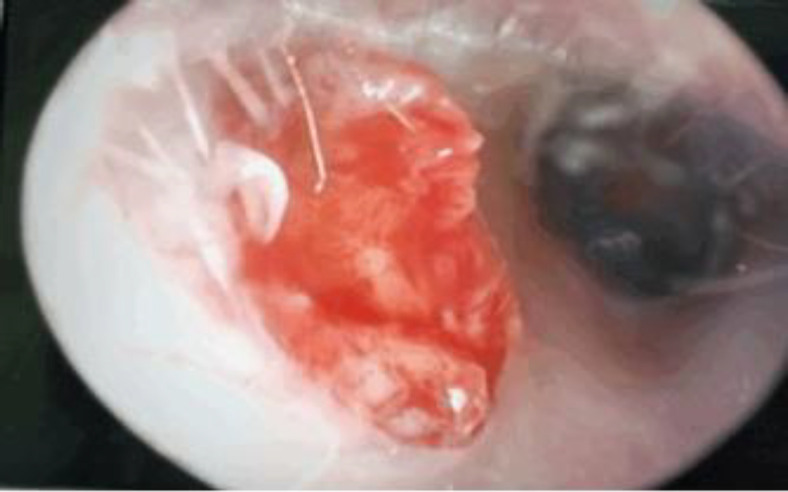
Tympanic membrane and the rest of the ear canal visualised upon excision of the granulation tissue

Since the swelling reduced with ear discharge, the granulation tissue (Figure 4) was excised and the pus was manually drained by daily massaging and milking from the openings of the fissure of Santorini. Pus was sent for culture and sensitivity to which later isolated no microorganism. In view of the clinical improvement from the intravenous antibiotics aided with drainage to the ear, the patient was treated successfully by nonsurgical means. Follow-up on the patient one month later showed that she is asymptomatic and examination revealed a normal ear canal with no opening or fibrosis on inspection.

## Discussion

Infectious and neoplastic matters are presumed to be travelling through the fissures of Santorini, however, the exact direction of its spread has yet to be reported. 

A number of studies had demonstrated that the spread of disease from the parotid gland extending to the ear was far more common in those of neoplastic origin as compared to the infectious origin (2-4). Most of the cases had history of ear trauma with fistula complication, few had been attributed to the naturally occurring tracts. Studies dated back to 2015 and 2017 by Onal et al and Blanco-Sanchez et al respectively, reported cases of parotid abscess manifesting in the form of otorrhea. Interestingly, patients were those of paediatric population*. *(5,6). 

While in elderly immunocompromised patient, infections such as malignant otitis externa has been reported to invade the parotid gland (7). Cholesteatoma is another type of infection that spread anteriorly and cause parotid abscess (8). No neoplastic case in this direction was reported. Parotid abscess can be managed conservatively or surgically. In a study of 40 patients, 82% of patients were managed conservatively (9). Small-sized collections can be treated medically with combination of antibiotics, hydration and sialogogues. In large collections, an incision is required to drain the affected space. There is risk of facial nerve damage and poor cosmetic results pertaining to this invasive treatment option (10). Apart from surgical incision, there are other alternatives for parotid abscess drainage. Percutaneous aspiration guided by ultrasonography has proven to be effective and of less invasive technique (11,12). Drainage can also be conducted by milking the abscess from the Stensen's duct opening (13). 

As for our case, we manage to avoid all the techniques above, also ensuring the maternal and fetal safety whilst treating the parotid abscess albeit at a slower pace. As the patients clinical condition greatly improved upon daily external compression of the parotid gland, we discovered a unique process of resolution of parotid abscess through an opening in the ear canal. This will not be a standard of treatment or a recommended technique. Although this is an unconventional method, it is noteworthy to mention that the drainage through the fissure of Santorini has proven to be an effective one. 

## Conclusion

In conclusion, drainage is the mainstay of treatment, however, after factoring in multiple variables and weighing the risks and the benefits, we managed the case uniquely, which proved effective. In spite of the fact that the fissure of Santorini is known as the gateway and tunnel for a disease to spread, it has proven to serve as a pathway for disease elimination as well.
